# Tumour invasion and dissemination

**DOI:** 10.1042/BST20220452

**Published:** 2022-06-17

**Authors:** Ryan Lusby, Philip Dunne, Vijay K. Tiwari

**Affiliations:** 1 Wellcome-Wolfson Institute for Experimental Medicine, School of Medicine, Dentistry and Biomedical Science, Queens University, Belfast BT9 7BL, U.K.; 2 Patrick G Johnston Centre for Cancer Research, Queen's University, Belfast BT9 7AE, U.K.

**Keywords:** cancer, cell shape, metastasis

## Abstract

Activating invasion and metastasis are one of the primary hallmarks of cancer, the latter representing the leading cause of death in cancer patients. Whilst many advances in this area have been made in recent years, the process of cancer dissemination and the underlying mechanisms governing invasion are still poorly understood. Cancer cells exhibit multiple invasion strategies, including switching between modes of invasion and plasticity in response to therapies, surgical interventions and environmental stimuli. The ability of cancer cells to switch migratory modes and their inherent plasticity highlights the critical challenge preventing the successful design of cancer and anti-metastatic therapies. This mini-review presents current knowledge on the critical models of tumour invasion and dissemination. We also discuss the current issues surrounding current treatments and arising therapeutic opportunities. We propose that the establishment of novel approaches to study the key biological mechanisms underlying the metastatic cascade is critical in finding novel targets that could ultimately lead to complete inhibition of cancer cell invasion and dissemination.

## Introduction

Tumour cell migration and invasion are the key drivers of metastatic dissemination, resulting in the development of metastatic tumours at secondary sites, and remains the primary cause of cancer-related death [1]. Activation of invasion and metastasis is one of the primary hallmarks of cancer and involves multiple processes, including changes in cell morphology, polarity and translocation of the cell body [2].

Invasion is one of the earliest steps in a cascade of phenotypic events that culminates in the metastatic dissemination of tumour cells. It involves the process of malignant cells detaching from the tumour mass, acquiring a plastic phenotype to actively move and invade the surrounding tissues.

**Figure 1.**
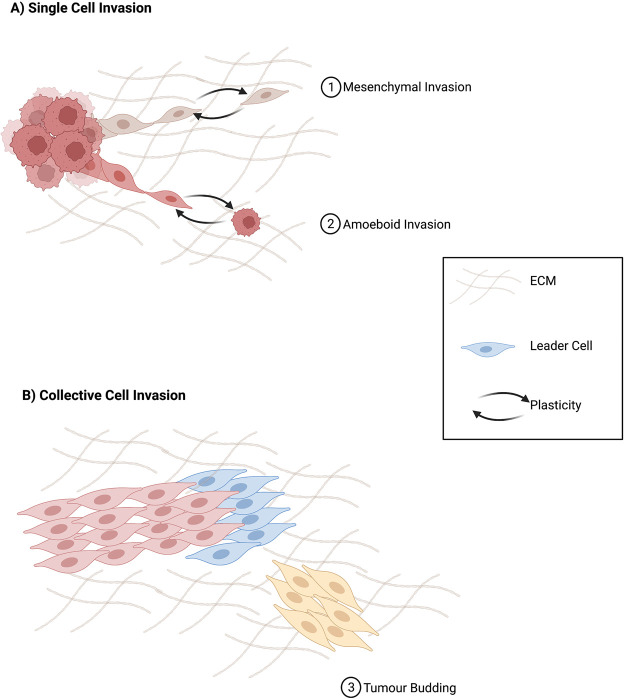
Invasion and dissemination of cancer cells. Diagram showing an overview of individual and collective invasion.

Cancer cells have been shown to be able to adapt to different stimuli from both the surrounding environment and therapeutic intervention. They have the exceptional ability to undergo invasion, dissemination and migrate in distinct modes, either individually or collectively. While single-cell migration is the primary mode of invasion into the vascular and lymphatic systems, collective migration is the primary form of invasion and dissemination in most solid tumours [3] (Figure 1).

Understanding the dynamics of invasion and dissemination in cancers will help identify biomarkers that could predict patients metastatic potential upfront and uncover novel targets for precision therapies that can disrupt key steps in the metastatic cascade ultimately preventing metastatic disease from progressing or possibly reversing metastatic cancer growth.

## Single-cell invasion vs collective cell invasion

Invasion of tumour cells is one of the earliest steps in the metastatic cascade. It can be characterised by either single cell or collective cell invasion. Single-cell invasion and dissemination frequently present as two distinct movement types, mesenchymal and amoeboid, based on unique and reversible morphological and expression patterns [4] (Figure 1). In mesenchymal invasion, degradation of the ECM is a critical and unique feature when compared with amoeboid migration [5,6]. Cancer cells undergoing mesenchymal invasion recruit proteases that actively remodel the ECM, enabling the generation of cell migration tracks by which mesenchymal cancers cells disseminate [5,6]. Recently, it has been shown targeted inhibition of these proteases can convert cells undergoing mesenchymal type invasion to an amoeboid type, highlighting their plasticity [7]. Amoeboid movement is defined due to the changes in cell shape and inherent plasticity in which they can move through the ECM without requiring its remodelling [8]. Additionally, they have been shown to move significantly faster in comparison with the mesenchymal type [9].

The key features defining collective cell invasion include the movement of cancer cells which remain physically grouped together through cell-to-cell junctions. The groups of cells that detach from the primary tumour have a leading-edge comprised of leader cells with mesenchymal phenotypes [10]. These leader cells actively remodel the ECM and drag follower cells, with epithelial phenotypes through the migration track in the ECM [11–13]. Tumour budding is a primary feature of collective invasion, routinely identifiable at the invasive front of tumours [14], and is regarded as an indicator of the onset of cancer invasion and metastasis and is associated with poor prognosis in various cancers [15–18].

E-cadherin is one of the critical markers of cell-to-cell adhesion and thus is found to have high expression in collective-cell invasion and is commonly down-regulated in single-cell invasion. In breast cancer, higher expression of E-cadherin was correlated with reduced invasion and increased metastasis due to the maintenance of cell-to-cell adhesions [19]. In collective invasion, cell-to-cell adhesion enables disseminating clusters of cells to migrate into the vascular system as circulating tumour cells (CTC) clusters. Detection of these cellular clusters within the circulation indicates a worse prognosis than detecting single CTCs by single-cell invasion [20].

## Role of EMT in single and collective invasion

The process of epithelial-mesenchymal transition (EMT) is thought to be a critical component that enables cancer cells to initiate invasion and dissemination [21]. EMT is the reversible process in which immotile epithelial cells, tightly bound with one another and the surrounding ECM, develop the ability to transition towards a more mesenchymal phenotype [22,23]. This epithelial-mesenchymal plasticity enables tumour cells to invade, acquire therapy resistance, and disseminate. EMT is induced by molecular changes in cancer cells and their secretion of cytokines and growth factors in the tumour microenvironment (TME) [24].

In the early stages of invasion and dissemination single tumour cells will lose cell-to-cell adhesion and undergo EMT [25]. Inducing EMT in tumour cells requires cross-talking with stromal cells, in particular with cancer-associated fibroblasts (CAFs). CAFs are one of the most abundant cell types within the TME, and higher levels of CAFs are associated with poor prognosis [26]. Although EMT is a programme that causes cells to transition from an epithelial phenotype to a mesenchymal phenotype, there is evidence that collective invading cells do not lose their epithelial phenotypes completely [19].

Tumour cell clusters in collective migration undergo a hybrid EMT process, characterised by the co-existence of epithelial and mesenchymal traits. Recently studies have shown that subpopulations of cancer cells associated with a hybrid EMT state have an advantageous ability for progressing with invasion and dissemination than a complete mesenchymal state and contribute to malignant phenotypes [27,28]. Additionally, the role of a hybrid EMT state as the central role of collective invasion has been supported by identifying core EMT transcription factors in multiple cancers. In pancreatic ductal adenocarcinoma, ZEB1 is expressed in the tumour bud, and head and neck squamous cell carcinoma SNAIL is critically involved in collective cell migration [29,30]. Furthermore, in cell line models of breast cancer, it has also been shown in leader cells with a hybrid EMT state are involved in collective invasion with high expression of core EMT transcription factors including TWIST-1, ZEB1 and ZEB2 [31,32]. Furthermore, a recent study has shown that the hybrid EMT state is acquired through stromal CAFs-mediated paracrine signalling through induction of ZEB1 [33].

Following EMT tumour cells have been shown to have further plasticity by gaining the ability to acquire amoeboid features and thus enhancing invasion and dissemination. It has shown to increase the invasiveness of many cancers [34]. For example in lymph node-negative breast cancers it has been shown to have a potential in assessing early stage metastatic risk [35].

Additionally, compared with the non-EMT cancer cells, cells with EMT phenotype have more developed anti-apoptotic systems and show more resistance to therapy [36]. Whilst EMT is involved in multiple stages of invasion and dissemination, it is a topic of great debate whether it plays a critical role in metastasis and chemotherapy resistance. In lung and pancreatic cancers, there is growing evidence that EMT might not be the main programme underlying metastasis, instead, it has been shown to be a key driver of chemoresistance in these cancers [37–40]. Amidst these questions around the critical role of EMT in the initiation of tumour dissemination, the opposite process of mesenchymal-epithelial transition (MET) is a vital component of metastatic progression and the development of metastatic tumour formation [41]. Nevertheless, further research is required to fully uncover the exact role of EMT entirely in cancer invasion, dissemination and chemoresistance.

## Extracellular matrix remodelling

The extracellular matrix (ECM) is the primary structural component of the tumour microenvironment, consisting of networks of interconnected macromolecules that are present in multiple tissue types and cancers [42]. However, the ECM primary role is not solely as structural support, it plays a critical role in cell–cell communication and invasion of tumour cells [43].

Cellular mechanosensing, in which cells identify mechanical signals, through the activation of mechanosensors, play a critical role in tumour cell invasion through the ECM [44]. Invading tumour cells favour a stiffer ECM, which is detected by mechanosensors, including integrins and focal adhesions (FA),to trigger a series of mechanotransductions [45–48]. For example, Durotaxis, a form of cell invasion in which the cell migration is directed by a gradient of ECM-stiffness, is thought to play a key role in EMT and tumour invasion [49,50]. Studying how tumour cells sense increased ECM stiffness and why they discriminately migrate within a stiffer ECM could result in a greater understanding of tumour cell invasion and the identification of novel therapeutics. A recent study developed a computation model of directed cell migration toward a stiffer ECM and found that they are primarily guided by filopodial mechanosensing. Additionally they highlighted that the abundance of short and abundant filopodia correlates with a more aggressive phenotype [51]. Fascin protein is the main actin-binding protein in filopodia and is elevated expression in metastatic tumours [52], and inhibition using fascin-specific small-molecules reduces tumour cell migration and tumour metastasis in mouse models [53].

In carcinoma *in situ*, tumour cells are prevented from invading into the surrounding tissue by a basement membrane. The ability for cancer cells to disseminate requires the degradation of the ECM by several ECM remodelling events. Proteolytic degradation is the primary step in breaking down the basement membrane and enabling invasion of surrounding tissue. This is achieved through the secretion of target-specific proteases such as matrix metalloproteinases (MMPs) and additional target-specific proteases. These target-specific proteases are significantly overexpressed in multiple cancers and are frequently correlated with worsened survival outcomes [54,55].

**Figure 2.**
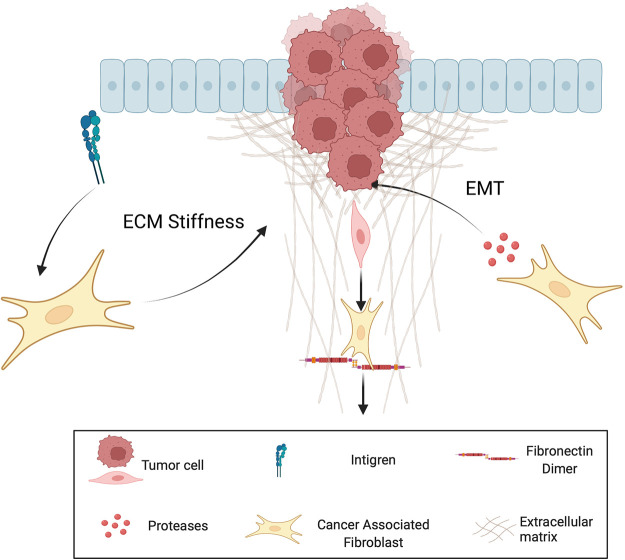
Role of cancer associated fibroblasts in ECM remodelling. Cancer associated fibroblasts induce EMT, facilate ECM stiffness, and create migrating tracts for invading cells.

CAFs are thought to act as leader cells of tumour cell invasion by clearing the ECM through proteolytic and force mediated remodelling processes [56] (Figure 2). CAFs have been shown to further drive MMP-independent invasion of tumour cells through the basement membrane [57]. Additionally CAFs interact with integrins and promote Rho-mediated regulation of myosin light chain activity to apply force to the ECM and align collagen fibres [58,59]. In particular, the Rho family of small guanosine triphosphatases (GTPases) have been shown to be involved in EMT and thus are critical for cell motility and facilitate the dissemination of tumour cells [60,61]. Furthermore, the binding of fibronectin to CAF integrins forces the self-assembly of dimers resulting in the opening of gaps in the ECM to further facilitate migration [62].

In contrast, amoeboid cells have the ability to pass through the ECM in the absence of ECM remodelling via proteolytic processes [34]. Amoeboid tumour cells exhibit bleb-like protrusions which enable faster movement due to the lack of adhesion [63]. ECM stiffness is has been observed to regulate the switch for mesenchymal to amoeboid migration and is facilitated by activation of the ROCK-myosin II pathway via Rho GTPase regulation [64].

The diversity in which tumour cells can invade and disseminate through the ECM enables them to retain their migratory ability across varying environmental pressure. Thus, targeted therapeutics and biomarkers which can detect and prevent ECM could hold the potential for improving patient outcomes.

## Role of nerves in the invasion and dissemination of cancer cells

The dissemination of solid tumours is historically classified by three main processes, direct invasion into surrounding tissue lymphatic and vascular systems. However, an additional function, the dissemination of cancer cells through nearby nerves, is less understood [65–68]. The TME plays a key role in cancer initiation, progression, and dissemination. Interestingly, nerves, consisting of various cells such as neurons and neuroglia, have been shown to emerge in the TME and the presence of which has been well-established for multiple cancers and has been shown to associate with significantly poorer outcomes [69–71].

In recent years perineural invasion (PNI) has increasingly to been associated as a significant pathological feature of many cancers and its presence is associated with worsened overall survival and disease-free survival in head and neck, prostate and pancreatic cancers [72–74]. The dissemination of cancer cells through this process is thought to occur through similar processes in which they move through the vascular and lymphatic systems. In which cancer cells migrate along and around nerves following infiltration into the perineural space [75]. Critically, this process has been identified before invasion into both the vascular and lymphatic systems [76,77]. Evidence of this movement has been detected in pancreatic ductal adenocarcinoma, where disseminating cancer cells were shown to migrate into the spinal cord along sensory neurons [65].

This additional mode of invasion and dissemination provides a unique opportunity to identify novel biomarkers which could detect metastatic cancer before invasion into the vascular and lymphatic systems. However, the understanding of crucial PNI mechanisms underlying invasion and dissemination remains limited, mainly due to a lack of an applicable model in which to explore and replicate the extensive interactions between tumour cells and nerves.

## Surgical intervention potentially initiates tumour invasion and dissemination

Surgical removal remains one of the primary methods to treat and control the progression of most solid tumours. Whilst surgical removal of primary tumours is typically associated with increased survival, the primary cause of metastasis following surgery is due to the presence of dormant cancer cells which have already migrated to secondary sites before surgery and evaded elimination by the patients’ immune system [78].

Additionally, there is evidence that following surgical excision cancer cells have the ability to survive by retaining their ability to invade leading to the acceleration of tumour recurrence. The unavoidable damage to the patients’ tissues during excision and manipulation of the tumour being resected and its vasculature have been shown to disseminate tumour cells into the blood and lymphatic circulation [79]. Circulating tumour cells (CTCs) in the blood are an indicator for diagnosis, prognosis, and therapeutic response in multiple cancers [80–83]. Following surgery, CTCs have been observed to increase and are associated with an increased chance of patients developing residual disease [84–87]. However, this needs to be further explored to determine the direct clinical relevance.

It has been suggested that the anaesthetics administered to patients during surgical excision can potentially increase the rate of metastasis. In ovarian, melanoma and colon cancers there have been contradictory findings with associating residual disease potential and whether they received general or localised anaesthetics [88,89]. These findings have also been shown to have similar outcomes in *in vitro* models of breast cancer, where the anaesthesia sevoflurane is associated with increased proliferation, migration and invasion [90,91].

Both experimental and clinical evidence supports the idea that surgery intended to be a curative option to remove and reduce tumour mass may unfortunately also increase invasion and dissemination of cancer cells. Suppose one can address those factors in the peri-operative period, which foster the capture and promotion of metastases. In that case, the immediate post-operative period may become a unique window to control and target residual malignant cells.

## Therapeutic interventions for metastatic disease

Despite extensive efforts to understand the key driving factors of tumour invasion, dissemination, and metastatic disease, the identification of metastatic sites continues to be associated with the worst possible outcomes for patients. Although prevention of invasion and dissemination has been demonstrated to have a benefit preclinically, the characterisation and development of novel therapeutics have been unsuccessful [92–94]. One of the key hurdles in designing metastatic treatments is patient selection in clinical trials. They are normally advanced staged metastatic and therapy-resistant patients, due to exhaustion of all other therapy options. Due to this, many therapies’ effectiveness cannot be tested in this short timeframe [95].

### Potential implications of chemotherapy in triggering invasion and dissemination

Unlike primary tumours, metastasis is a systemic disease, where tumours cells have usually already disseminated to secondary sites [93]. To date, there is a lack of targeted therapies which account for this systemic issue and have ultimately been unable to prevent or reverse metastatic progression in patients. Unfortunately surgical excision of the secondary tumours having little benefit in patient outcomes, so metastatic patients are subjected to treatment regimens that are aimed to control further metastatic spread through the administration of systemic treatments including chemotherapy, radiotherapy and immunotherapy [96].

Whilst chemotherapy has been shown to have a great clinical utility in the treatment of primary tumours, this cannot be said for the treatment of metastatic disease, even for chemotherapy-treated patients with control of the primary tumour [97]. This issue could be in part related due to the pre-existence of chemoresistant clones which following chemotherapy remain and have the distinct ability to metastasise to distant sites and propagate the growth of chemoresistant metastatic tumours [98–100]. Additionally, whilst the TME plays a key role in each stage of invasion and dissemination of tumour cells it also has been shown to have a fundamental role in chemotherapy resistance [101–103]. In particular, following chemotherapy, specific subsets of resistant cancer cells can persist and expand, driving disease progression. [98]. Resistant cells features are largely overlapping with the phenotype and properties of cancer stem cells (CSCs) including self-renewal ability, metastatic capability and cell plasticity [104,105]. EMT can be considered the link between chemoresistance and metastatic potential in this context. However, this connection might be more complex than initially imagined, and several aspects still need to be thoroughly investigated [106]. Besides the direct cytotoxic or damaging effect on tumour cells, or indirect anti-tumour immune stimulatory effects resulting from cells undergoing multiple forms of cell death, chemotherapy may also induce host-mediated pro-metastatic changes through systemic release of cytokines and chemokines, mimicking an injury-like response as typically detected in wound healing and inflammation processes [107,108]. This release of chemokine/cytokines, for example by VEGFR-1 expressing endothelial cells in lung cancer [109], is thought to initiate the expansion of a subset of non-canonical regenerative CSCs that can promote tumour relapse and stimulate metastasis-receptive niches by establishing an environment of supportive stromal cells at distant sites [108,110]

Accumulating pre-clinical evidence suggests that chemotherapy can disrupt each step of the metastatic cascade and induce intra-tumoral and systemic changes that can promote cancer cell survival/proliferation, ultimately fostering dissemination to distant organs [108,111,112]. In a recent study it was shown that neoadjuvant chemotherapy increases the intravasion of tumour cells. Groups of macrophages, endothelial and tumour cells, termed TME of metastasis, where shown to enable the movement of tumour cells into the vasculatory system and where elevated following chemotherapy [113]. An additional mechanism in which chemotherapy can contribute to metastasis is through the increased expression of Lysyl oxidase (LOX) in CD8+ T cells. In mouse models of breast cancer, it was recently shown that expression of LOX in CD8+ T cells resulted in ECM remodelling in the lungs and enabled seeding for circulating tumour cells [114]. Interestingly they have shown that inhibiting LOX reverses the increased risk of metastatic tumour formation following chemotherapy, highlighting that a greater understanding of the key mechanisms which drive metastasis hold the potential for treating the disease.

One of the critical unmet needs in cancer therapy is the treatment of metastatic disease. Due to metastatic disease being associated with chemoresistance it is vital to gain an understanding of the key mechanisms underlying invasion and dissemination following treatment. At the same time, the identification and validation of predictive biomarkers for high risk chemoresistant and metastatic patients is vital to progress personalised treatments and improve clinical outcomes.

### Current efforts to target tumour invasion and dissemination

Whilst the therapeutic benefits of chemotherapy are well documented they have been shown to have paradoxical effects in the treatment of metastatic cancer, however there are few alternatives in the clinical setting. Thus, the current challenge to unravel the key mechanisms in which chemotherapy resistance and metastasis develop is fundamental in the development of strategies to improve chemotherapy response and target or reverse metastatic disease.

Multiple studies have focussed on attempting to disrupt the pathways driving tumour invasion and dissemination. However, inhibition of a single pathway ultimately leads to resistance [115]. Matrix metalloprotease inhibitors have been shown to prevent mesenchymal types of migration but unfortunately could not prevent invasion overall [116]. Although resistance of singular inhibition can be explained due to the redundancy of many intracellular signalling processes, there remains the potential for precise targeting of novel pathways [117]. Such as Cyclin-Dependent Kinase 4/6 (CDK4/6) Inhibitors which have been shown to have a promising clinical outcomes in breast cancer [118]. Additionally in metastatic prostate cancer, androgen receptor inhibitors in metastatic prostate cancer, have been shown to be positively correlated with survival. However, unfortunately in metastatic breast cancer, they have had little improvements towards patient survival [119,120].

Compared with single pathway inhibition, it has been demonstrated that targeting multiple pathways simultaneously seems vital in countering the significant features of metastatic tumour cells [121]. For example in HER2 positive metastatic breast cancer patients, a combination treatment of tucatinib, trastuzumab, and capecitabine has been reported to improve patient outcomes [122]. Additionally, in melanoma patients with metastatic disease, a combination immunotherapy of Nivolumab and ipilimumab has been shown to have significantly positive effect on clinical outcomes [123].

**Figure 3.**
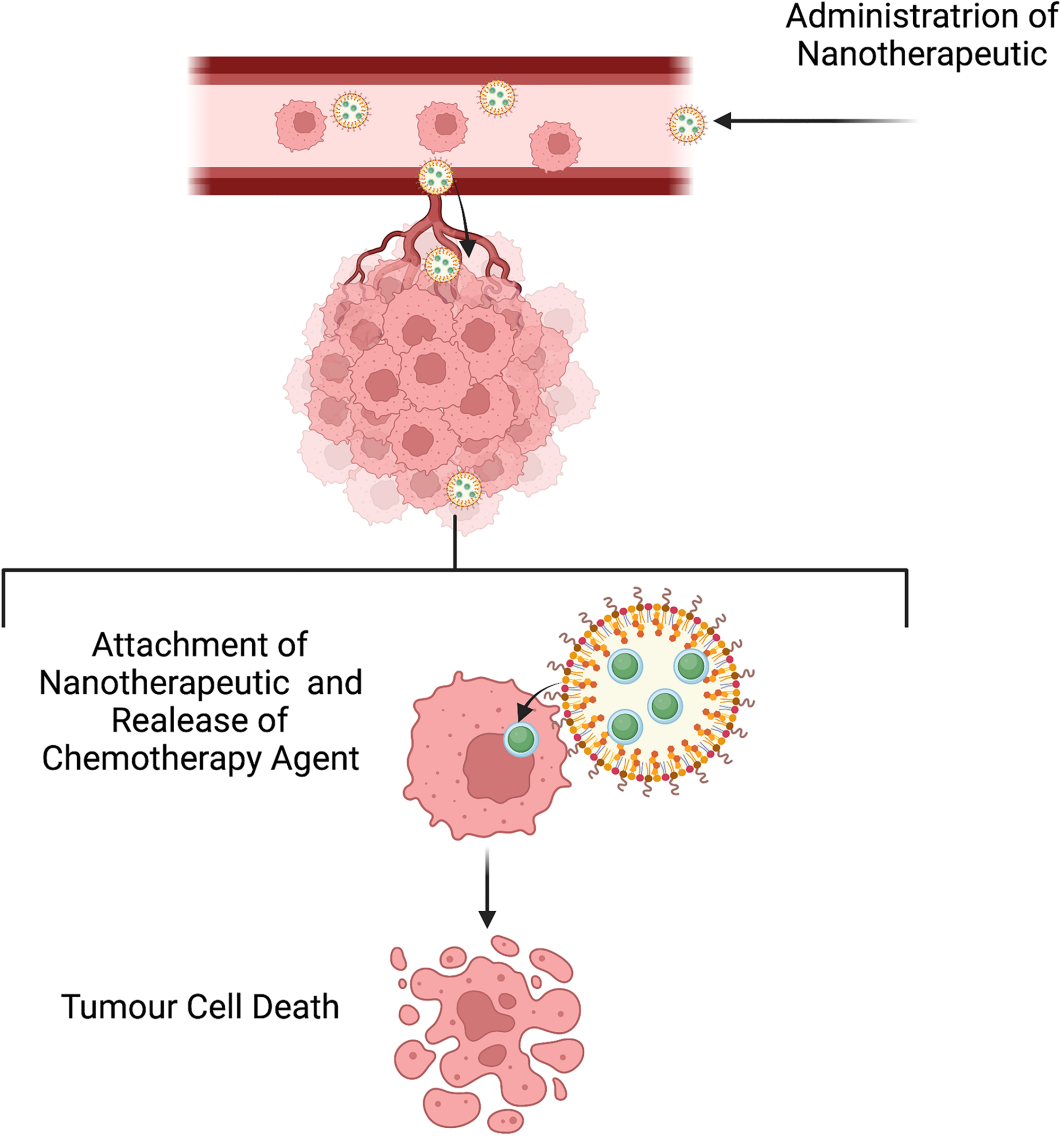
Nanotheraputics in primary and metastatic cancer. Nanotherapeutic containing chemotherapy which following entry into the vascular system can target primary and metastatic tumour cells resulting in targeted cell death.

In recent years nanotechnology-based approaches hold a significant promise in the improvement of anti-cancer and anti-invasion therapies [124]. In particular, the advent of nanotherapeutics have the potential to overcome many of disadvantages of chemotherapy and traditional therapeutic modalities by encapsulating anti-cancer agents and enable site specific targeting of primary and metastatic tumours [125] (Figure 3). In hepatocellular carcinoma a MRI visable Non-Coding-RNA-based EMT/CSC Inhibitory nanotherapeutic designed to target STAT3, successfully inhibited tumour growth, invasion, and migration [126]. Additionally, in cervical cancer a Nanoquinacrine not only reduced the invasion and proliferation of CSCs, but also sensitises 5-FU resistant CSCs [127].

The combination of nanotheraputics and chemotherapy have been shown to overcome chemotherapy resistance and metastasis in breast cancer cell lines. The theranostic nanocomposite (Ag-TF@PDOX), consisting of silver nanoparticles and doxorubicin, has been shown to increase cytotoxicity. Additionally, not only did it reverse chemotherapy resistance it also revers metastasis at the subcellular level. It primary achieves this through the down-regulation of P-glycoprotein via an increase in ATP-consuming chaperones [128].

Overall, tumour invasion and dissemination is a complex challenge, highlighted by despite extensive study, there have been no approved targeted therapies to prevent or reverse metastatic disease. The identification of biomarkers that could potentially predict early-stage cancer patients metastatic potentially could offer a unique opportunity to target invasion and dissemination before metastatic disease develops through the use of nanotherapeutics.

## Conclusions

The migration and metastasis of cancer cells to peripheral sites remain the primary cause of cancer-related deaths [129]. Whilst Chemotherapy is the standard treatment for many patients with metastatic cancer it too can elicit negative consequences such as chemoresistance and pro-metastatic responses. Owing to the complexity of metastatic disease, a complete understanding of the molecular mechanisms which underly invasion and dissemination remains a significant challenge. Due to in part there are a lack prospective studies with long patient follow-up and current *in vitro* methods cannot replicate the metastatic process efficiently [130,131].

A more comprehensive analysis of the underlying mechanisms and long-term response to current treatments is paramount to enable the identification of predictive biomarkers for therapy response and metastatic potential. This greater understanding will enable the identification of high-risk patients at earlier disease stages and may enable the identification of novel therapeutics to overcome resistance and reverse or prevent the progression of metastatic disease.

## Perspectives

- Tumour cell migration and invasion is the key driver of metastatic dissemination and the primary cause of death in cancer.
- The key biological underpinnings which govern tumour invasion and dissemination is currently lacking, resulting in a lack of targeted therapeutics and biomarkers which can treat and detect metastatic disease in its early stages. Chemotherapy remains the standard treatment whilst the potential for immunotherapy and targeted nanotherapeutics to treat metastatic disease are still a main focus of research.
- Novel methods to explore the evolution of tumour invasion and dissemination are required to enable better characterisation and assessment of therapeutic interventions. Additionally, future development of nanotherapeutics hold a great potential in targeted treatment of primary and metastatic tumours.

## Abbreviations

CAFs: cancer-associated fibroblasts
CSCs: cancer stem cells
CTC: circulating tumour cells
ECM: extracellular matrix
EMT: epithelial-mesenchymal transition
LOX: Lysyl oxidase
PNI: perineural invasion
TME: tumour microenvironment
